# An Objection to “Listerism”*Read before the Buffalo Medical and Surgical Association.

**Published:** 1882-06

**Authors:** C. M. Daniels


### An Objection to "Listerism."*

By C. M. Daniels, M. D.

Much has been said for and against the so-called "Listerism," or antiseptic spray, used and advocated by gentlemen high in authority and strongly combatted by others having equal opportunities to judge of its merits. * * * It is not my intention to try to advance any new ideas in antiseptic surgery, nor to pretend to speak authoritatively upon that subject. But as many of the

*Read before the Buffalo Medical and Surgical Association.

papers which have been read before this society have so fully exhausted their several subjects, that there has been little to comment upon, or to draw forth much discussion or criticism, I venture to present this subject this evening, expecting it will at least offer a subject for discussion.

The point taken, is in regard to the spray, as used in ovariotomy, or any operation where an elevated temperature is, or is supposed to be, required. Although such elevation is not conceded as necessary by some authorities, I believe none recommend that it should be below 80°. My attention was first called to the peculiar coldness of the spray while preparing the atomizer for use; and to satisfy myself as to the decrease in temperature, caused apparently by the rapid evaporation, I instituted a series of experiments to thoroughly test the variations practically, leaving theory entirely out of the question. I obtained a reliable ten inch thermometer and prepared several antiseptic solutions as hereafter described, using a room with a constant temp. of 100°. First experiment, used water in cup of atomizer at 58°; spray thrown upon thermometer at a distance of 26 inches reduced temp. to 73° in five and to 70° in ten minutes, where it remained stationary. Next, used a solution of carbolic acid 1 to 20 at 58° which reduced the temp. to 73° in five and to 67° in ten minutes, showing a wider variation by 3° than with water alone. I then heated the carbolic solution to 180°, but it produced precisely the same result. Then a solution of salicylic acid 1 to 150 was tried, and produced the same result as the carbolic. Finally a solution of thymol at 180° was tried under the same conditions, and which reduced the temp. to 78° in five and to 74° in ten minutes; this was repeated with solution at 60°, but without change. This shows (perhaps in opposition to theory) that the thymolic solution evaporates less rapidly than either of the others stated. Not feeling entirely satisfied with my experiments as described, and fearing that there might have been influences exerted that I did not fully understand, at the suggestion of a medical friend, I obtained permission of the proprietor of the Turkish bath rooms to continue my experiment there under entirely different conditions and influences, as well as in a much higher temperature, and wishing some one else to witness it, I was accompanied by Dr. A. M. Barker. We entered the room where the temperature was 135° dry heat, as shown by two thermometers, at which point it remained for one hour and ten minutes, the time consumed in the test. A carbolic solution of 1 to 20 was used as before, and the spray directed upon one thermometer at a distance of two feet. In one minute it registered 112°, in three minutes 105° and in six minutes 100½°, where it remained stationary for twenty minutes, thus showing a reduction of 34½°, or an incease of 1½° over the same solution tried in a temp. of 100°, evidently showing that the greater the elevation, the greater is the proportionate reduction. This I further demonstrated by using the same solution in a temperature of 80°, and could only reduce it to 68° in half an hour, this not being as low as when the room was at 100°.

I am fully aware that these experiments were performed in a crude manner, and do not claim exactness, as certain conditions might possibly cause variations of a degree or more. But the idea I think is fully carried out, and I shall feel well repaid if this will receive enough attention to induce others to investigate further. Now, the inference naturally gained by this would seem to be that it must be productive of much harm to throw a spray at these temperatures over an opening into the abdominal cavity, as the operations are often prolonged, and sitting aside the question of irritation caused by the spray (as claimed by many) it would seem that the greater danger lies in exposing the peritoneum, while in a healthy condition, to continued cold. In my experience with our lamented friend, Prof. White, covering twenty cases of ovariotomy, I can confidently assert that the best results were obtained where the spray was not used, as the average temperature of patients during the first twenty-four hours was lower and recoveries more rapid. * * *

